# Phenotype-based modeling of postoperative ventilatory liberation failure: A dynamic mechanistic framework for precision perioperative medicine

**DOI:** 10.1016/j.jatmed.2026.06.004

**Published:** 2026-07-28

**Authors:** Mohammed Said Bouya, Hamza Najout, Najib Bouhabba

**Affiliations:** a Department of Anesthesiology and Critical Care, Oued Eddahab Military Hospital, Ibn Zohr University, Agadir 80000, Morocco; b Department of Anesthesiology and Critical Care, Mohammed V Military Teaching Hospital, Mohammed V University, Rabat 10100, Morocco

**Keywords:** Postoperative extubation failure, Ventilatory liberation, Diaphragm-protective ventilation, Respiratory drive, Cardiovascular load intolerance, Precision perioperative medicine

## Abstract

Postoperative ventilatory liberation failure is usually recorded as a late binary event, although vulnerability often develops earlier through anesthesia, mechanical ventilation, neuromuscular blockade, analgesia, fluid therapy, surgical stress, and the transition to spontaneous breathing. This conceptual review proposes a phenotype-based framework that redefines postoperative liberation failure as a dynamic syndrome generated by four interacting dimensions: neuromuscular contractile insufficiency, effort-related diaphragmatic myotrauma, central respiratory-drive suppression, and cardiovascular load intolerance. Using conceptual framework analysis and a scoping-informed mapping strategy, the model links cumulative perioperative exposures to stage-specific vulnerability trajectories spanning readiness assessment, spontaneous-breathing-trial stress, and post-extubation sustainability. The framework is not a validated bedside algorithm, but a falsifiable mechanistic architecture for phenotype definition, prospective validation, mechanism-directed trials, and interpretable clinician-in-the-loop decision support.

## Introduction

Failure to liberate a postoperative patient from invasive mechanical ventilation represents a major inflection point in the trajectory of care. Extubation failure, delayed extubation, and unplanned reintubation are associated with pneumonia, diaphragmatic deconditioning, cardiovascular decompensation, prolonged intensive care, and mortality; yet these outcomes are usually documented only after the physiological opportunity for prevention has partially passed.^1^, ^2^

Traditional classifications of weaning failure were largely derived from medical intensive care populations and typically focus on broad categories such as difficult or prolonged weaning. These categories are useful for epidemiology but insufficient for perioperative medicine, where risk is created by temporally dense and often reversible exposures: anesthetic depth, neuromuscular blockade, opioids, surgical inflammation, pain, atelectasis, fluid balance, hemodynamic stress, body position, and the abrupt transition from positive-pressure ventilation to spontaneous breathing.

Ventilatory liberation should therefore not be viewed as a single functional test performed at time t, but as the measurable downstream expression of cumulative exposures affecting the diaphragmatic effector, central neural drive, patient-ventilator synchrony, and cardiovascular tolerance to spontaneous breathing. This view is aligned with the emerging concept of lung- and diaphragm-protective ventilation, which recognizes that both excessive unloading and injurious respiratory effort can harm the respiratory pump.^3^, ^4^

This review proposes a phenotype-based conceptual framework for postoperative ventilatory liberation failure. Its purpose is not to provide a validated bedside algorithm, but to establish a foundational mechanistic architecture that can organize perioperative physiology, guide measurement, structure future trials, and support interpretable artificial-intelligence models. The central thesis is that liberation failure becomes scientifically tractable only when the endpoint is decomposed into falsifiable phenotypes, mapped to time-dependent exposures, and tested against reproducible markers.

## Conceptual framework development

### Design and reporting logic

This work was conceived as a structured theoretical model rather than a conventional narrative review. The primary method was Jabareen’s conceptual framework analysis, which is suited to complex phenomena with key constructs spanning heterogeneous clinical, physiological, and technological literatures.^5^ To increase transparency, the framework was reinforced by a scoping-informed mapping strategy aligned with the Preferred Reporting Items for Systematic reviews and Meta-Analyses extension for Scoping Reviews (PRISMA-ScR) and Joanna Briggs Institute (JBI) guidance.^6^, ^7^

### Search strategy and eligibility criteria

The search strategy targeted perioperative liberation failure, respiratory muscle biology, respiratory drive, cardiovascular intolerance to spontaneous breathing, and point-of-care physiological monitoring. The core PubMed/MEDLINE query was: (“Ventilator Weaning”[Mesh] OR “Airway Extubation”[Mesh] OR liberation OR “spontaneous breathing trial” OR reintubation) AND (“Postoperative Period”[Mesh] OR anesthesia OR anaesthesia OR perioperative OR surgery) AND (“Diaphragm”[Mesh] OR “respiratory drive” OR “neuromuscular blockade” OR “patient-ventilator asynchrony” OR “pulmonary edema” OR “lung ultrasound” OR echocardiography). Searches were supplemented by backward and forward citation tracking of major guidelines, mechanistic reviews, diagnostic accuracy studies, and trials relevant to the four candidate domains. The search was run from database inception to April 2026 and updated in May 2026.

Eligible sources included clinical studies, randomized or observational perioperative studies, systematic reviews, mechanistic intensive care unit (ICU) physiology studies, neuromuscular monitoring guidelines, ultrasound studies, ventilatory interaction studies, and conceptual papers that could inform postoperative liberation mechanisms. Exclusion criteria were pediatric-only studies unless they provided uniquely relevant mechanistic information, animal-only studies without direct translational relevance, non-English articles without an accessible abstract, isolated chronic respiratory disease studies without perioperative or liberation relevance, and purely technical engineering papers without bedside physiological interpretation. Because the objective was framework construction, methodological quality was considered during interpretation but was not used as the sole reason for excluding conceptually informative sources.

### Concept extraction, synthesis, and saturation

The working corpus comprised 134 publications selected for conceptual relevance to perioperative liberation, diaphragmatic function, residual neuromuscular blockade, respiratory drive, ventilator-induced diaphragm dysfunction, lung and diaphragm ultrasound, cardiovascular weaning failure, perioperative ventilation trials, and machine-learning prediction. Selection was intentionally theory-building rather than prevalence-estimating; therefore, the corpus should be interpreted as a conceptual evidence map, not as a formal systematic review denominator. To increase methodological transparency, this work is reported as a conceptual synthesis and not as a systematic or scoping review: no formal dual independent screening, risk-of-bias scoring, or PRISMA-style flow diagram was applied. Source identification and concept extraction were performed primarily by the lead author and were iteratively discussed and adjudicated by all authors until conceptual saturation, with methodological quality considered interpretively rather than as a sole exclusion criterion. A conceptual evidence map linking the retained sources to the four mechanistic dimensions and the three liberation stages is provided in Supplementary.

The analytic procedure comprised: (1) mapping of heterogeneous data sources; (2) categorization of entities related to liberation failure, including ventilator-induced diaphragmatic dysfunction (VIDD), residual neuromuscular blockade, patient-ventilator asynchrony, respiratory drive suppression, and weaning-induced pulmonary edema (WIPO); (3) identification and naming of latent concepts; (4) deconstruction of overlapping concepts; (5) integration into mechanistically coherent categories; (6) synthesis into a limited set of dimensions; (7) theoretical validation against physiological and clinical evidence; and (8) refinement by introducing temporal sequencing, dimensional overlap, reversibility, and counterexamples.

A central methodological decision was to separate mechanisms that are often grouped under the umbrella of diaphragmatic dysfunction. VIDD may include disuse atrophy caused by excessive unloading, but respiratory muscle injury may also result from excessive or dysynchronous loading. These mechanisms are physiologically distinct and may require opposite ventilatory responses; therefore, they were treated as separate conceptual dimensions rather than as a single diagnostic category.^3^, ^4^

The decision to retain exactly four primary dimensions was guided by parsimony and by the anatomy of the respiratory pump pathway. Successful liberation requires that an adequate central command (drive) reach a competent effector (neuromuscular contractility), generate force within a non-injurious range of mechanical load (effort), and be tolerated by the cardiovascular system once positive-pressure support is withdrawn (cardiovascular load tolerance). These four nodes are sequential, individually perturbable, mechanistically non-redundant, and frequently call for opposite corrective actions—for example, increasing versus reducing ventilatory assistance—so that collapsing them into a single “diaphragmatic dysfunction” category would obscure clinically decisive distinctions. Each dimension is also independently falsifiable, which served as the criterion for admitting a candidate as a primary domain rather than as a modifier, boundary condition, or competing outcome (Section Falsifiability, boundary conditions, and a possible fifth dimension).

### Dynamic vulnerability formalization

To make the temporal dimension explicit, liberation vulnerability may be represented conceptually as a time-dependent function: $V(t)=B_{0}+\sum P+\int_{0}^{t}\left[N\left(t\right)+E\left(t\right)+C\left(t\right)+H(t)\right]\mathrm{dt}+I(t)$, where $B_{0}$ denotes baseline reserve (incorporating age, frailty, sarcopenia, and cardiopulmonary reserve), ∑Pcumulative perioperative exposures, $N\left(t\right)$ neuromuscular vulnerability, $E\left(t\right)$ effort-related loading, $C\left(t\right)$ central-drive suppression, H(t) hemodynamic/cardiovascular vulnerability expressed as load intolerance, and I(t) interaction terms between dimensions. The formula is not intended for immediate clinical calculation; it formalizes the hypothesis that vulnerability emerges from cumulative dose, timing, reversibility, and cross-domain amplification. This expression is intended as an illustrative heuristic rather than a directly measurable equation; in its present form the variables are not assigned units, weights, or fixed thresholds. Operationalization would require expressing each domain term (*N*, *E*, *C*, *H*) as a standardized, scaled vulnerability index derived from its candidate markers, estimating the relative weights and the interaction terms I(t) empirically in prospective postoperative cohorts, and validating the resulting score against adjudicated liberation outcomes through the staged research programme outlined in Section Translational research agenda: from concept to foundational programme. Until such validation exists, V(t) should be read as a conceptual organizing device, not as a bedside calculator.

The model also separates three decision stages that are often conflated: Stage 1, readiness to attempt liberation; Stage 2, tolerance of the spontaneous breathing trial (SBT) as a physiological stress test; and Stage 3, post-extubation sustainability, including airway protection, respiratory reserve, secretion clearance, analgesia-sedation balance, and cardiovascular tolerance. This staged view is essential for developing future predictive models, because a patient may successfully pass Stage 2 yet fail Stage 3 through mechanisms that were not stressed or measured during the SBT.^8^

## Results: phenotype-based modeling of postoperative liberation failure

### Directed graph structure

**Fig. 1:**
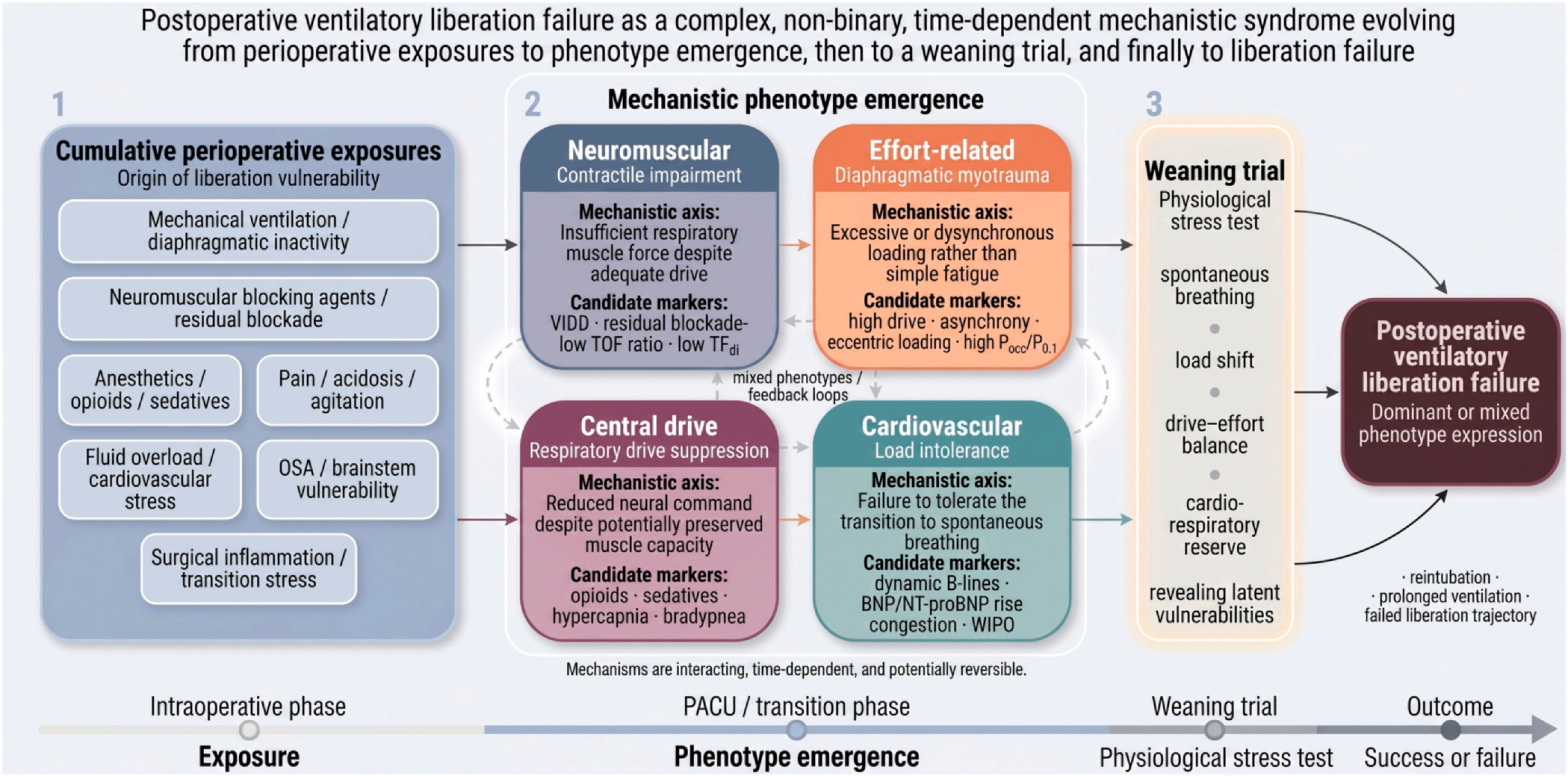
Phenotype-based conceptual framework of postoperative ventilatory liberation failure. Cumulative perioperative exposures may generate liberation vulnerability before any formal weaning attempt. During the post-anesthesia care unit transition, this vulnerability may emerge through four interacting mechanistic phenotypes: neuromuscular contractile impairment, effort-related diaphragmatic myotrauma, central respiratory-drive suppression, and cardiovascular load intolerance. The weaning trial functions as a physiological stress test that challenges spontaneous breathing, load shift, drive-effort balance, and cardiorespiratory reserve, thereby revealing latent vulnerabilities. The bottom timeline should be interpreted in parallel with the dynamic vulnerability function V(t): Stage 1 corresponds to readiness and latent vulnerability before formal testing, Stage 2 to SBT stress, and Stage 3 to post-extubation sustainability or failure. Solid arrows indicate putative mechanistic progression, whereas dashed arrows indicate mixed phenotypes, feedback loops, time-dependent interactions, and potential reversibility. The terminal outcome reflects dominant or mixed phenotype expression. This figure is a theoretical conceptual framework and does not constitute a validated clinical algorithm. To aid interpretation, the four phenotype nodes should be read as potentially co-existing rather than mutually exclusive: a patient may move between nodes along the dashed arrows (for example, a central-drive phenotype converting into an effort-related phenotype as PaCO_2_ rises during the SBT), and the dominant node may differ at Stage 1, Stage 2, and Stage 3. Abbreviations: BNP, B-type natriuretic peptide; NT-proBNP, N-terminal pro-B-type natriuretic peptide; OSA, obstructive sleep apnea; PACU, post-anesthesia care unit; *P*_0.1_, airway occlusion pressure measured 100 ms after inspiratory occlusion; *P*occ, airway occlusion pressure during brief end-expiratory occlusion; SBT, spontaneous breathing trial; TFdi, diaphragm thickening fraction; TOF, train-of-four; VIDD, ventilator-induced diaphragmatic dysfunction; WIPO, weaning-induced pulmonary edema. Phenotypes may coexist; the dominant phenotype may change across Stages 1–3; and Stage 3 includes airway protection, secretion clearance, delirium, and competing postoperative pulmonary complication pathways.

The framework articulates four dominant mechanistic dimensions within a directed graph that links perioperative exposures, phenotype emergence, the weaning trial, and the terminal clinical outcome (Fig. 1). The graph is explanatory rather than prescriptive: arrows indicate putative causal relationships, mechanistic overlap, mixed phenotypes, dynamic feedback loops, and time-dependent reversibility rather than fixed diagnostic categories.

### Neuromuscular dimension: contractile impairment

The neuromuscular dimension describes an intrinsic or extrinsic inability of the diaphragm and associated respiratory muscles to generate sufficient tension despite an otherwise appropriate central drive. It is dominated by two mechanisms with different kinetics: disuse atrophy and residual neuromuscular blockade.

Perioperative diaphragmatic inactivity can rapidly trigger oxidative stress, proteolytic activation, impaired protein synthesis, mitochondrial dysfunction, and sarcomeric injury, contributing to VIDD.^4^, ^9^, ^10^ In parallel, residual neuromuscular blockade may impair inspiratory force, upper-airway patency, swallowing, and protective reflexes, particularly when quantitative neuromuscular monitoring and complete reversal are not systematically applied.^11^, ^12^ Candidate empirical markers include low diaphragm thickening fraction (TFdi), reduced tidal volume generation, weak maximal inspiratory pressure, and incomplete train-of-four (TOF) recovery. Baseline contractile reserve is further shaped by age, frailty, and sarcopenia, which reduce both diaphragmatic and limb-muscle capacity and lower the threshold at which any phenotype produces liberation failure; frailty is therefore treated here as a cross-cutting baseline modifier (a determinant of *B*_0_) rather than as a separate primary dimension.^13^

For research purposes, a conservative TOF threshold of at least 0.95 can be used to minimize residual blockade misclassification and isolate non-pharmacological contractile impairment. However, this threshold should be framed as a research normalization target, not as an established clinical mandate. Emerging device-based strategies such as non-invasive magnetic phrenic nerve stimulation are now being tested specifically to preserve diaphragm thickness in early mechanically ventilated patients, providing an intervention-level anchor for the neuromuscular dimension while awaiting efficacy results.^14^

### Effort-related dimension: diaphragmatic myotrauma

The effort-related dimension is distinct from simple fatigue. It reflects injurious loading of the respiratory muscles caused by excessive drive, under-assistance, patient-ventilator asynchrony, or eccentric diaphragmatic contraction. In this phenotype, the diaphragm attempts to shorten while mechanical conditions force lengthening or disproportionate load, creating conditions for myofibrillar injury and inflammatory signalling.^3^, ^4^

In practical terms accessible to the anesthesiologist, effort-related diaphragmatic myotrauma is best understood as load-induced muscle injury rather than energetic fatigue. When respiratory drive is high and assistance is insufficient or poorly synchronized, the diaphragm develops large transdiaphragmatic pressures and may be forced to contract while lengthening (eccentric or “pendelluft” loading); these high local stresses disrupt sarcomeres and activate inflammatory and proteolytic signalling, and they can paradoxically coexist with preserved or even increased muscle thickness. This is the mechanistic mirror image of the disuse atrophy described above, which is precisely why the neuromuscular and effort-related dimensions are kept separate: one reflects too little loading of the muscle, the other too much.

Perioperative triggers include abrupt transitions in ventilatory support, inadequately treated pain, acidosis, agitation, anxiety, secretion burden, atelectasis, or dyssynchronies such as ineffective efforts and reverse triggering. Candidate markers include severe tachypnea, accessory muscle recruitment, high airway occlusion pressure measured 100 ms after inspiratory occlusion (*P*_0.1_) or end-expiratory occlusion pressure (*P*_occ_), high asynchrony burden, increasing respiratory rate-to-tidal volume ratio, and dynamic exhaustion of TFdi during the effort period.^15^, ^16^, ^17^

Proportional modes—such as neurally adjusted ventilatory assist (NAVA) and proportional assist ventilation plus (PAV+) —may be mechanistically attractive in high-drive or highly asynchronous patients because they adjust assistance to patient effort. A recent randomized postoperative trial in major abdominal surgery patients undergoing hepatic procedures reported more favorable weaning physiology with NAVA compared with pressure support, supporting this dimension as a testable perioperative strategy while not yet proving broad outcome benefit.^18^

### Central drive dimension: respiratory drive suppression

The central drive dimension occurs when the respiratory muscles may remain structurally capable, but the neural command generated or integrated by the central nervous system is insufficient. It may result from residual anesthetic agents, opioids, sedatives, metabolic alkalosis, sleep-disordered breathing phenotypes, brainstem vulnerability, or postoperative delirium with alternating agitation and suppression.

This phenotype is suggested by hypoventilation without proportional distress, bradypnea, hypercapnia, high end-tidal carbon dioxide, reduced diaphragmatic excursion despite preserved end-expiratory thickness, and temporal association with sedatives, opioids, sleep-disordered breathing, or residual anesthetic effect. Nociception-monitoring signals such as Nociception Level index (NOL), Analgesia Nociception Index (ANI), Surgical Pleth Index (SPI), or pupillometry may help quantify analgesic exposure and avoid opioid over- or under-treatment, but they should not be interpreted as validated markers of respiratory-drive suppression; current evidence supports cautious outcome-oriented use rather than device-driven causal labeling.^19^

The central phenotype is clinically important because escalating mechanical assistance alone may mask rather than correct the underlying problem. A phenotype-directed response would prioritize sedative minimization, opioid-sparing analgesia, reversal of reversible depressant effects when appropriate, correction of metabolic contributors, noninvasive support for sleep-disordered breathing phenotypes, and continuous capnographic surveillance during the vulnerable transition period.

### Cardiovascular dimension: load intolerance

The cardiovascular dimension corresponds to the inability of the postoperative heart and pulmonary circulation to tolerate the transition from positive-pressure ventilation to spontaneous breathing. WIPO reflects the combined effects of increased venous return, increased left ventricular afterload, altered ventricular interdependence, catecholaminergic activation, ischemia or myocardial injury, and an overloaded or diastolically vulnerable ventricle.

Candidate markers include dynamic development of lung ultrasound B-lines, elevated or rising B-type natriuretic peptide (BNP) or N-terminal pro-BNP (NT-proBNP), reactive hypertension, oxygenation deterioration, and echocardiographic signs of diastolic or systolic vulnerability. In postsurgical patients, a > 23.3% rise in NT-proBNP during a two-hour SBT was associated with weaning failure and showed moderate discrimination, supporting biomarker dynamics as an enrichment signal rather than as a stand-alone decision rule.^20^

Individualized positive end-expiratory pressure (PEEP) regimens may attenuate the cardio-respiratory burden of the transition to spontaneous breathing. In the perioperative setting, a PEEP decrement trial following a recruitment maneuver has been shown to improve oxygenation and lung mechanics in selected settings.^21^, ^22^ Ongoing flow-controlled ventilation trials in robot-assisted laparoscopy may further clarify whether intraoperative ventilatory strategies can reduce postoperative pulmonary vulnerability before the liberation window.^23^

### Phenotype identification matrix and falsifiable propositions

Table 1 summarizes candidate empirical markers and corresponding theoretical propositions. The numerical values are not clinical recommendations; they are candidate markers derived largely from critical care physiology, perioperative monitoring, and ultrasound literature, and require prospective validation in strictly postoperative cohorts.

**Table 1:** Candidate empirical markers and related theoretical propositions.

**Dominant dimension**	**Underlying pathophysiology**	**Candidate empirical markers (to validate)**	**Associated theoretical propositions**
Neuromuscular	Disuse atrophy, VIDD, residual neuromuscular blocking-agent effect, or acquired respiratory-muscle weakness.	Quantitative TOF ratio < 0.9; research normalization target ≥ 0.95; TFdi < 26–30%; reduced diaphragmatic excursion; weak MIP; low tidal-volume generation; longitudinal diaphragm-thickness loss. (Quantitative neuromuscular monitoring and confirmed TOF recovery before extubation = E; TFdi cut-offs, the ≥0.95 normalization target, and MIP/excursion thresholds = H.)	P1: Mandatory quantitative neuromuscular monitoring and reversal, combined with diaphragm ultrasound, will reduce misclassified neuromuscular phenotypes and identify residual VIDD after pharmacologic causes have been excluded.
Effort-related	Excessive respiratory drive, under-assistance, dyssynchrony, eccentric contraction, pain-driven tachypnea, acidosis, and effort-related diaphragmatic myotrauma.	*P*_0.1_/*P*_occ_ > 3.5–4 cmH_2_O; high respiratory rate; asynchrony index > 10%; accessory-muscle recruitment; waveform signs of ineffective effort or double triggering; dynamic fall in TFdi; pain/acidosis trajectories. (Asynchrony and accessory-muscle recruitment are observable signs (E); all numerical cut-offs (*P*_0.1_/*P*_occ_ >3.5–4 cmH_2_O, asynchrony index >10%) are H.)	P2: In high-drive postoperative patients, phenotype-triggered proportional assistance such as NAVA or PAV + will reduce asynchrony and injurious effort compared with fixed pressure support.
Central drive	Residual anesthetics or opioids, sedative accumulation, sleep-disordered breathing phenotype, metabolic suppression, brainstem vulnerability, or excessive assistance masking intrinsic drive.	Rising EtCO_2_ or PaCO_2_; bradypnea; low diaphragmatic excursion despite preserved thickness; hypoventilation without proportional distress; opioid/sedative burden; cautious use of nociception-monitor trajectories as exposure markers. (Rising EtCO_2_/PaCO_2_ and bradypnea are evidence-supported signs of hypoventilation (E); their attribution to central-drive suppression, and the use of nociception-monitor trajectories as drive markers, = H.)	P3: In patients with central suppression defined by hypercapnia, reduced excursion, and preserved contractile reserve, prioritized reduction or reversal of respiratory depressants will restore drive more effectively than escalation of ventilatory assistance alone.
Cardiovascular	WIPO caused by preload/afterload shift, diastolic vulnerability, myocardial injury, pulmonary vascular disease, venous congestion, or fluid overload.	Dynamic lung-ultrasound ΔB-lines; rising BNP or NT-proBNP, including ΔNT-proBNP > 23.3% during SBT as a candidate enrichment marker; reactive hypertension; oxygenation deterioration; echocardiographic congestion or load intolerance. (Dynamic B-lines and a rising NT-proBNP are evidence-associated with weaning failure (E for association); the ΔNT-proBNP > 23.3% cut-off and its use as a stand-alone rule = H.)	P4: Individualized PEEP and fluid/afterload optimization guided by mechanics, lung-heart ultrasound, and biomarker dynamics will reduce expression of a congestive phenotype compared with fixed PEEP in patients at cardiovascular risk.

Evidence status of markers: items are tagged E (evidence-supported) or H (hypothesis-generating and requiring prospective postoperative validation); all numerical cut-offs in this table are H. Abbreviations: ANI, Analgesia Nociception Index; BNP, B-type natriuretic peptide; EtCO_2_, end-tidal carbon dioxide; MIP, maximal inspiratory pressure; NAVA, neurally adjusted ventilatory assist; NOL, Nociception Level index; NT-proBNP, N-terminal pro-BNP; *P*_0.1_, airway occlusion pressure measured 100 ms after inspiratory occlusion; PAV+, proportional assist ventilation plus; PEEP, positive end-expiratory pressure; *P*_occ_, airway pressure generated during end-expiratory occlusion; SPI, Surgical Pleth Index; SBT, spontaneous breathing trial; TFdi, diaphragm thickening fraction; TOF, train-of-four; VIDD, ventilator-induced diaphragmatic dysfunction; WIPO, weaning-induced pulmonary edema.

## Discussion

### Operationalizing diaphragm-protective perioperative ventilation

The main value of this framework is that it provides a mechanistic architecture for diaphragm-protective ventilation in the perioperative setting. For many years, ventilatory protection focused primarily on the lung, with attention to volutrauma, barotrauma, atelectrauma, driving pressure, and oxygen toxicity. The present model adds the respiratory pump as an explicit therapeutic target.

The neuromuscular and effort-related dimensions represent two opposite poles of diaphragmatic harm. Excessive unloading may promote disuse atrophy, whereas excessive or dysynchronous loading may promote myotrauma. A diaphragm-protective strategy should therefore avoid both extremes and aim to maintain effort within a physiologically acceptable range. Candidate signals such as *P*_0.1_, *P*_occ_-derived estimates of muscle pressure, respiratory rate, tidal volume pattern, asynchrony burden, and diaphragm ultrasound may help titrate assistance, although perioperative thresholds require prospective validation.^3^, ^15^, ^16^, ^17^

A substantial part of the supporting physiology—including *P*_0.1_ and *P*_occ_ thresholds, TFdi cut-offs, the ventilator-induced diaphragm dysfunction literature, and proportional-mode and NT-proBNP data—originates from medical intensive care unit weaning cohorts rather than from postoperative surgical populations. Translation to the perioperative setting cannot be assumed, because these populations differ in ways that are likely to shift every threshold: postoperative ventilation is usually short and elective rather than prolonged, sedation and opioid exposure follow an anesthetic rather than a critical-illness trajectory, the systemic inflammatory burden is typically lower and more transient, patient selection and comorbidity profiles differ, and extubation is frequently planned in the operating room or PACU rather than after a protracted weaning course. Consequently, ICU-derived cut-offs should be regarded as starting hypotheses requiring recalibration—and possibly re-derivation—in strictly postoperative cohorts before any bedside use.

The framework also does not require the simultaneous availability of every monitoring modality. A pragmatic, tiered approach can be proposed: a minimum set feasible in most settings—structured clinical assessment, quantitative train-of-four monitoring, capnography, and basic lung-ultrasound B-line assessment—supports a simplified bedside triage of the dominant phenotype, whereas advanced or optional tools—diaphragm thickening fraction, *P*_0.1_/*P*_occ_, echocardiography, and NT-proBNP dynamics—are reserved for enriched or high-risk patients and for research validation. Framed in this way, the model degrades gracefully: where advanced physiology is unavailable, a clinician can still identify the most likely and most reversible dimension from widely accessible signals.

### Temporal vulnerability trajectories and mixed phenotypes

The formal function *V*(t) emphasizes that liberation failure depends not only on the presence of a risk factor but also on its timing, cumulative burden, interaction with other domains, and reversibility. A patient with obesity and obstructive sleep apnea may enter the postoperative period with a central drive vulnerability that is amplified by opioids. During an SBT, hypoventilation, atelectasis, and rising carbon dioxide may then increase respiratory drive and secondarily convert a central phenotype into an effort-related phenotype. More specifically, central hypoventilation raises PaCO_2_, which stimulates respiratory centres and may transform an initially depressive phenotype into excessive respiratory effort during the SBT. This trajectory is different from a patient whose first dominant abnormality is residual neuromuscular blockade or from a patient whose SBT immediately unmasks diastolic load intolerance.

Mixed phenotypes are therefore expected rather than exceptional after major surgery. A patient may simultaneously have partial residual neuromuscular blockade, fluid overload, pain-driven high respiratory effort, and opioid-related hypoventilation. The clinical value of the framework lies less in assigning a single label than in identifying the dominant and most reversible mechanism at a given time point. Highly reversible contributors such as residual blockade, excessive opioid effect, undertreated pain, fluid overload, and severe dyssynchrony should be corrected first, followed by reassessment of phenotype dominance.

#### Illustrative vignette

Consider an older patient with obesity, obstructive sleep apnea, and mild frailty undergoing prolonged upper-abdominal surgery. At Stage 1 (readiness), reduced baseline reserve (*B*_0_) and residual neuromuscular blockade dominate, so quantitative train-of-four monitoring with complete reversal is the priority. At Stage 2 (SBT), opioid-related hypoventilation initially suggests a central-drive phenotype, but the rising PaCO_2_ drives a compensatory increase in effort, and an effort-related pattern with high *P*_0.1_ and asynchrony emerges. At Stage 3 (post-extubation sustainability), intraoperative fluid loading unmasks a cardiovascular load-intolerance phenotype with new B-lines and a rising NT-proBNP, while sedation-related delirium and secretion burden threaten airway protection. No single label applies throughout; the value of the framework is to identify, at each stage, the dominant and most reversible mechanism to correct first.

### Evidence anchoring and validation roadmap

Table 2^11^, ^12^, ^14^, ^15^, ^16^, ^17^, ^18^, ^19^, ^20^, ^21^, ^22^, ^23^, ^24^, ^25^, ^26^, ^27^, ^28^, ^29^, ^30^ links each theoretical proposition to empirical evidence anchors, residual uncertainty, and a practical validation design. This table is deliberately conservative: published evidence supports the plausibility of the framework, but few studies have tested postoperative phenotype-guided liberation as an integrated intervention. Recent protocols and trials should therefore be used to sharpen falsifiable propositions, not to overstate clinical validation.

**Table 2:** Theoretical propositions linked to empirical evidence anchors, residual uncertainty, and practical validation designs for postoperative ventilatory liberation.

**Proposition**	**Evidence anchor**	**Critical uncertainty**	**Most appropriate validation design**
P1: Neuromuscular normalization	ASA and ESAIC guidelines support quantitative neuromuscular monitoring and TOF recovery before extubation; diaphragm ultrasound meta-analyses support TFdi as a specific but incompletely sensitive marker of weaning risk; the STIMIT ACTIVATOR 1 protocol provides an intervention-level test of diaphragm-thickness preservation.^11^, ^12^, ^14^, ^15^	TOF normalization does not eliminate airway, pharyngeal, central drive, or cardiovascular causes of failure. The postoperative value of a stricter TOF ≥ 0.95 target and device-based diaphragm stimulation remains unproven.	Prospective perioperative cohort or cluster trial using mandatory quantitative monitoring, standardized reversal, diaphragm ultrasound, and adjudicated failure phenotypes; pilot interventional studies testing diaphragm-preservation strategies in enriched high-risk patients.
P2: Effort-related myotrauma prevention	Respiratory-drive physiology and *P*_0.1_/*P*_occ_ studies support non-invasive estimation of drive and effort; proportional modes may improve synchrony; a recent postoperative NAVA trial in major abdominal surgery supports feasibility and physiological plausibility.^15^, ^16^, ^17^, ^18^, ^24^, ^25^, ^30^	Thresholds for harmful postoperative effort are unknown. It is unclear whether proportional assistance improves hard outcomes beyond short-term synchrony or ventilatory duration.	Randomized physiological trial comparing phenotype-triggered proportional assistance with conventional pressure support in high-drive postoperative patients, with effort, asynchrony, TFdi, dyspnea, and extubation outcomes as endpoints.
P3: Central-drive restoration	Opioid/sedative exposure, sleep-disordered breathing, capnography, hypercapnic trajectories, and cautious use of nociception-monitor outputs such as the Nociception Level index (NOL), Analgesia Nociception Index (ANI), Surgical Pleth Index (SPI), or pupillometry provide plausible exposure markers for central-drive suppression.^19^, ^26^	No validated perioperative phenotype definition separates central suppression from neuromuscular weakness, excessive assistance, or airway obstruction. Proprietary nociception indices do not directly measure respiratory neural output.	Prospective diagnostic study using capnography, diaphragm ultrasound, structured sedation/opioid exposure, and non-invasive nociception-monitor trends (Nociception Level index (NOL), Analgesia Nociception Index (ANI), Surgical Pleth Index (SPI), or pupillometry) as exposure signals, followed by response to opioid-sparing or reversal strategies.
P4: Cardiovascular load-intolerance prevention	Lung ultrasound and combined lung-heart-diaphragm ultrasound studies support dynamic congestion assessment during SBT; postsurgical NT-proBNP change during SBT shows moderate discrimination; individualized PEEP improves perioperative mechanics in selected contexts.^20^, ^21^, ^22^, ^23^, ^27^, ^28^, ^29^	Whether mechanics-, biomarker-, or ultrasound-guided PEEP prevents WIPO during postoperative liberation is untested. NT-proBNP dynamics require external validation and should not be used as a stand-alone rule.	Enriched randomized trial in patients with diastolic risk, fluid overload, myocardial injury, or ΔNT-proBNP rise, comparing fixed PEEP with individualized PEEP, fluid/afterload optimization, and ultrasound-guided reassessment.

Abbreviations: ANI, Analgesia Nociception Index; ASA, American Society of Anesthesiologists; BNP, B-type natriuretic peptide; ESAIC, European Society of Anaesthesiology and Intensive Care; NAVA, neurally adjusted ventilatory assist; NOL, Nociception Level index; NT-proBNP, N-terminal pro-BNP; *P*_0.1_, airway occlusion pressure measured 100 ms after inspiratory occlusion; PAV+, proportional assist ventilation plus; PEEP, positive end-expiratory pressure; *P*occ, airway pressure generated during end-expiratory occlusion; SBT, spontaneous breathing trial; SPI, Surgical Pleth Index; TFdi, diaphragm thickening fraction; TOF, train-of-four; WIPO, weaning-induced pulmonary edema.

### Falsifiability, boundary conditions, and a possible fifth dimension

A conceptual model belongs to the scientific domain only if it can be challenged by observations that contradict it. Following Popper’s principle of falsifiability, the strength of this framework depends not merely on its ability to organize known mechanisms, but on its vulnerability to counterexamples.^31^ The propositions P1–P4 are therefore framed as testable hypotheses rather than as clinical recommendations.

A potential falsifying scenario would be a postoperative patient who fails liberation despite complete normalization across all four proposed dimensions: quantitative neuromuscular transmission is confirmed, central drive is appropriate, diaphragm structure and thickening are preserved, and there is no evidence of dynamic pulmonary congestion. Such a patient would force expansion or revision of the framework.

This apparent framework-exceeding phenotype may reveal mechanisms not captured by the model, such as severe central airway collapse, tracheomalacia, occult fixed airway obstruction, inflammatory myopathy with initially preserved diaphragmatic thickness, or microcirculatory dysfunction of respiratory muscles not detected by thickness or thickening fraction.^32^, ^33^, ^34^, ^35^, ^36^ As a purely speculative future direction, it remains possible that a fifth dimension—respiratory muscle perfusion or oxygen-utilization failure—may eventually prove necessary. Emerging optical techniques, such as near-infrared spectroscopy of extradiaphragmatic inspiratory muscles, could eventually test this hypothesis. However, it falls outside the scope of the current four-dimension framework and is raised here solely to maintain the model’s openness to empirical revision.^35^, ^36^

It is therefore useful to state explicitly how several clinically important contributors relate to the four primary dimensions. Upper-airway obstruction, pharyngeal dysfunction, and airway edema are treated as boundary conditions and Stage 3 (post-extubation) airway-protection modifiers; mechanistically, several are partly downstream of the neuromuscular dimension, because residual neuromuscular blockade impairs pharyngeal function and predisposes to inspiratory upper-airway collapse.^37^, ^38^ Secretion burden and ineffective cough are likewise Stage 3 sustainability modifiers that determine whether a patient who passes the SBT can protect and clear the airway after extubation.^39^ Delirium-related behavioral factors are modeled as Stage 3 behavioral modifiers that interact with—and often amplify—the central-drive dimension rather than constituting a distinct mechanism of respiratory-pump failure.^40^ Postoperative pulmonary complications that are not primarily driven by diaphragm dysfunction (for example, pneumonia, atelectasis, or pleural effusion) are treated as a parallel, competing outcome pathway—one capable of causing liberation failure independently of the four phenotypes, and explicitly outside the scope of the present framework.^41^ Finally, frailty, sarcopenia, and reduced physiological reserve act as cross-cutting baseline modifiers embedded in *B*_0_. In each case the contributor was assigned to a modifier, boundary-condition, or competing-outcome role—rather than promoted to a fifth primary dimension—because it either lacks an independent, separately falsifiable mechanism of respiratory-pump failure or is better represented as a determinant of baseline reserve or post-extubation sustainability.

### Algorithmic modeling and artificial intelligence

The framework may also support future artificial-intelligence applications. Recent approaches to weaning and extubation prediction suggest that continuous physiological signals, ventilator waveforms, clinical trajectories, and ultrasound-derived variables may improve risk estimation beyond static bedside indices.^30^, ^36^, ^42^ A recently published Stage 3 extubation model within a three-stage liberation framework reported high discrimination for extubation success using routinely available electronic-record variables, but its low negative predictive value underscores the need for careful calibration, external validation, and phenotype-aware interpretation.^8^

A phenotype-based ontology can help annotate data, separate mechanisms, and avoid purely black-box classification. To date, however, no study has shown that phenotype-based annotation improves predictive performance compared with existing clinical prediction models; this is offered as a hypothesis to be tested, not as an established result. In this role, artificial intelligence would not replace clinician judgment or prescribe extubation decisions. Instead, it could provide calibrated risk estimates, display risk trajectories, identify probable risk-driving domains, and support prospective hypothesis testing within a clinician-in-the-loop framework. Rare-event prediction should report calibration, decision-curve analysis, and precision-recall metrics, and future studies should follow TRIPOD + AI to minimize reporting bias, information leakage, and non-transportable performance claims.^43^ Realizing this role will require overcoming substantial obstacles, including consistent ground-truth labeling of phenotypes, external and multicenter validation, prospective calibration, and the human-factors and governance barriers to clinical adoption.

### Translational research agenda: from concept to foundational programme

For the framework to become field-defining rather than merely descriptive, it must be converted into a staged research programme. The first phase should establish reproducible phenotype definitions and interobserver reliability. The second phase should test whether early perioperative markers predict phenotype emergence. The third phase should evaluate phenotype-triggered interventions in enriched populations. The fourth phase should embed the ontology into transparent decision-support systems and assess clinical utility, safety, and implementation fidelity (Table 3).

**Table 3:** Four-phase research roadmap for validating the perioperative liberation phenotype framework.

**Phase**	**Primary objective**	**Candidate outputs**
Phenotype reliability	Test whether clinicians and ultrasound/ventilator reviewers can consistently classify the four dimensions using prespecified signals.	Consensus definitions; adjudication manual; interobserver agreement; minimum dataset for postoperative liberation studies.
Temporal prediction	Determine whether intraoperative and PACU exposures predict phenotype emergence during SBT and after extubation.	Exposure-response models; dynamic vulnerability trajectories; candidate enrichment criteria for trials.
Mechanism-directed intervention	Test whether correcting the dominant reversible phenotype improves physiological and clinical outcomes.	P1–P4 pilot trials; adaptive platform design; phenotype-specific safety endpoints.
Decision support	Embed the ontology into electronic records or ventilator dashboards while maintaining clinician-in-the-loop governance.	Externally validated models; calibration and decision-curve analysis; prospective silent validation; implementation and drift monitoring.

This sequence avoids a common translational error: moving directly from plausible mechanisms to algorithmic prediction without validating the biological labels. The framework should therefore be treated as a bridge between perioperative physiology, pragmatic trial design, and interpretable artificial intelligence, not as a finished clinical pathway. In this sense, the proposed framework is intended to function as a foundational programme statement for perioperative liberation science rather than as another descriptive classification.

### Limitations

This review has several limitations. First, the framework is conceptual and scoping-informed rather than a completed systematic review or meta-analysis; therefore, it cannot quantify pooled effects, prevalence, or diagnostic accuracy across postoperative populations. Second, the corpus was selected for conceptual relevance, and residual selection bias is possible. Third, several candidate thresholds, including TFdi cut-offs, *P*_0.1_/*P*_occ_ ranges, ΔB-line values, ΔNT-proBNP dynamics, and stricter TOF normalization targets, were derived from heterogeneous ICU or perioperative physiology studies and require validation in strictly postoperative cohorts.

Fourth, some of the most promising emerging anchors are protocols, single-center studies, or early translational signals rather than definitive outcome trials. They were therefore integrated as hypothesis-generating evidence, not as proof of clinical effectiveness. Fifth, the framework has not yet undergone formal external Delphi validation. Future work should include an international panel of anesthesiologists, intensivists, respiratory physiologists, physiotherapists, nurses, and ultrasound experts to test construct clarity, face validity, feasibility, and global transportability.

Sixth, the four dimensions may not capture rare airway, neuromuscular, microcirculatory, metabolic, or neurological causes of liberation failure. The framework should therefore remain open to revision when counterexamples emerge. Its value is not that it closes the field, but that it creates a disciplined way to discover where the field is still incomplete.

## Conclusions

Postoperative ventilatory liberation failure should no longer be interpreted as the fatalistic endpoint of a single disconnection test. It is better understood as the clinical expression of a dynamic syndrome shaped by cumulative perioperative exposures, interacting physiological vulnerabilities, and stage-specific stressors that unfold from the operating room to the PACU, ICU, SBT, and post-extubation period.

By defining four falsifiable dimensions—neuromuscular contractile insufficiency, effort-related diaphragmatic myotrauma, central-drive suppression, and cardiovascular load intolerance—this phenotype-based framework provides a foundational architecture for perioperative diaphragm-protective ventilation and precision liberation research. More importantly, it establishes a translational research programme: first defining reproducible phenotypes, then validating stage-specific markers, then testing mechanism-directed interventions, and finally embedding the ontology into interpretable clinician-in-the-loop decision support.

## CRediT authorship contribution statement

**Mohammed Said Bouya:** Writing – review & editing, Writing – original draft, Resources, Methodology, Investigation, Formal analysis, Data curation, Conceptualization. **Hamza Najout:** Writing – review & editing, Software, Methodology. **Najib Bouhabba:** Visualization, Validation, Supervision, Project administration, Methodology, Conceptualization. All authors have read and agreed to the published version of the manuscript.

## Ethical statement

Ethics approval and informed consent were not required because this article is a conceptual review and did not involve human participants, human tissue, animal experiments, or identifiable individual patient data.

## Funding

This research did not receive any specific grant from funding agencies in the public, commercial, or not-for-profit sectors.

## Data availability

No new datasets were generated or analyzed during the preparation of this manuscript. All sources discussed are available in the published literature.

## Declaration of generative AI and AI-assisted technologies in the writing process

During the preparation of this work, the authors used AI-assisted language tools to improve readability, grammar, and editorial clarity. After using these tools, the authors reviewed and edited the content as needed and take full responsibility for the content of the published article.

## Declaration of competing interest

The authors declare that they have no known competing financial interests or personal relationships that could have appeared to influence the work reported in this paper.

## Footnotes

Appendix A Supplementary data associated with this article can be found in the online version at doi:10.1016/j.jatmed.2026.06.004.

## References

[1] Thille A.W., Richard J.C., Brochard L. (2013). The decision to extubate in the intensive care unit. Am J Respir Crit Care Med.

[2] Boles J.M., Bion J., Connors A. (2007). Weaning from mechanical ventilation. Eur Respir J.

[3] Goligher E.C., Dres M., Patel B.K. (2020). Lung- and diaphragm-protective ventilation. Am J Respir Crit Care Med.

[4] Fu W., Guan L., Liu Q. (2025). Ventilator-induced diaphragmatic dysfunction: pathophysiology, monitoring and advances in potential treatment and prevention. Eur Respir Rev.

[5] Jabareen Y. (2009). Building a conceptual framework: philosophy, definitions, and procedure. Int J Qual Methods.

[6] Tricco A.C., Lillie E., Zarin W. (2018). PRISMA Extension for Scoping Reviews (PRISMA-ScR): checklist and explanation. Ann Intern Med.

[7] Peters M.D.J., Marnie C., Tricco A.C. (2020). Updated methodological guidance for the conduct of scoping reviews. JBI Evid Synth.

[8] Chen C.M., Shao Y.C., Liu C.F. (2026). AI prediction of extubation success within a novel three-stage liberation framework: development, validation, and implementation of the Stage-3 model. Front Med (Lausanne).

[9] Levine S., Nguyen T., Taylor N. (2008). Rapid disuse atrophy of diaphragm fibers in mechanically ventilated humans. N Engl J Med.

[10] Goligher E.C., Dres M., Fan E. (2018). Mechanical ventilation-induced diaphragm atrophy strongly impacts clinical outcomes. Am J Respir Crit Care Med.

[11] Thilen S.R., Weigel W.A., Todd M.M. (2023). 2023 American Society of Anesthesiologists Practice Guidelines for Monitoring and Antagonism of Neuromuscular Blockade: A Report by the American Society of Anesthesiologists Task Force on Neuromuscular Blockade. Anesthesiology.

[12] Fuchs-Buder T., Romero C.S., Lewald H. (2023). Peri-operative management of neuromuscular blockade: a guideline from the European Society of Anaesthesiology and Intensive Care. Eur J Anaesthesiol.

[13] Gómez-Ríos M.Á., Abad-Gurumeta A. (2022). Anesthesia in the elderly patient. Resilience in frailty time. Med Clin (Barc).

[14] Schreiber A.F., Subirà C., Sklar M. (2025). Multi-center randomized superiority clinical trial in the early phase of mechanically ventilated patients to preserve diaphragm thickness using non-invasive magnetic phrenic nerve stimulation: STIMIT ACTIVATOR 1 pivotal trial. Trials.

[15] Le Neindre A., Philippart F., Luperto M. (2021). Diagnostic accuracy of diaphragm ultrasound to predict weaning outcome: a systematic review and meta-analysis. Int J Nurs Stud.

[16] Jonkman A.H., de Vries H.J., Heunks L.M.A. (2020). Physiology of the respiratory drive in ICU patients: implications for diagnosis and treatment. Crit Care.

[17] Telias I., Junhasavasdikul D., Rittayamai N. (2020). Airway occlusion pressure as an estimate of respiratory drive and inspiratory effort during assisted ventilation. Am J Respir Crit Care Med.

[18] Ibrahim E.S., Attalah H.A., Eid E.A. (2025). Neurally adjusted ventilatory assisted ventilation compared to pressure support during post-operative weaning of hepatic patients undergoing major abdominal surgeries: a randomized control trial. BMC Anesthesiol.

[19] Snoek M.A.J., van den Berg V.J., Dahan A. (2025). Comparison of different monitors for measurement of nociception during general anaesthesia: a network meta-analysis of randomised controlled trials. Br J Anaesth.

[20] Zheng Y., Luo Z., Cao Z. (2023). NT-proBNP change is useful for predicting weaning failure from invasive mechanical ventilation among postsurgical patients: a retrospective, observational cohort study. BMC Anesthesiol.

[21] Ferrando C., Mugarra A., Gutierrez A. (2014). Setting individualized positive end-expiratory pressure level with a positive end-expiratory pressure decrement trial after a recruitment maneuver improves oxygenation and lung mechanics during one-lung ventilation. Anesth Analg.

[22] Fernandez-Bustamante A., Sprung J., Parker R.A. (2020). Individualized PEEP to optimise respiratory mechanics during abdominal surgery: a pilot randomized controlled trial. Br J Anaesth.

[23] ClinicalTrials.gov. PROtective Ventilation With FLOW-Controlled Ventilation to Improve Postoperative Pulmonary Outcome After ROBOT-Assisted Laparoscopic Surgery: an international multicenter pilot randomized clinical trial (PROFLOW-ROBOTic). ClinicalTrials.gov identifier: NCT06703814. Accessed 6 May 2026.

[24] Umbrello M., Antonucci E., Muttini S. (2022). Neurally adjusted ventilatory assist in acute respiratory failure: a narrative review. J Clin Med.

[25] Arellano D.H., Brito R., Morais C.C.A. (2023). Pendelluft in hypoxemic patients resuming spontaneous breathing: proportional modes versus pressure support ventilation. Ann Intensive Care.

[26] Aishah A., Eckert D.J. (2019). Phenotypic approach to pharmacotherapy in the management of obstructive sleep apnoea. Curr Opin Pulm Med.

[27] Corradi F., Brusasco C., Vezzani A. (2016). Computer-aided quantitative ultrasonography for detection of pulmonary edema in mechanically ventilated cardiac surgery patients. Chest.

[28] Zhang Y., Chen J., Li X. (2024). Diagnostic accuracy of lung ultrasound to predict weaning outcome: a systematic review and meta-analysis. Front Med (Lausanne).

[29] Song J., Luo Q., Lai X. (2024). Combined cardiac, lung, and diaphragm ultrasound for predicting weaning failure during spontaneous breathing trial. Ann Intensive Care.

[30] Le Marec J., Hajage D., Decavele M. (2024). High airway occlusion pressure is associated with dyspnea and increased mortality in critically ill mechanically ventilated patients. Am J Respir Crit Care Med.

[31] Popper K.R. (1959). The Logic of Scientific Discovery.

[32] Murgu S.D., Colt H.G. (2006). Tracheobronchomalacia and excessive dynamic airway collapse. Respirology.

[33] Mariampillai K., Granger B., Amelin D. (2018). Development of a new classification system for idiopathic inflammatory myopathies based on clinical manifestations and myositis-specific autoantibodies. JAMA Neurol.

[34] Istfan R., Gómez C.A., Applegate M. (2021). Hemodynamics of the sternocleidomastoid measured with frequency domain near-infrared spectroscopy towards non-invasive monitoring during mechanical ventilation. Biomed Opt Express.

[35] Van Hollebeke M.V., Poddighe D., Clerckx B. (2022). High-intensity inspiratory muscle training improves scalene and sternocleidomastoid muscle oxygenation parameters in patients with weaning difficulties: a randomized controlled trial. Front Physiol.

[36] Park J.E., Kim D.Y., Park J.W. (2023). Development of a machine learning model for predicting weaning outcomes based solely on continuous ventilator parameters during spontaneous breathing trials. Bioengineering (Basel).

[37] Eikermann M., Vogt F.M., Herbstreit F. (2007). The predisposition to inspiratory upper airway collapse during partial neuromuscular blockade. Am J Respir Crit Care Med.

[38] Sundman E., Witt H., Olsson R. (2000). The incidence and mechanisms of pharyngeal and upper esophageal dysfunction in partially paralyzed humans: pharyngeal videoradiography and simultaneous manometry after atracurium. Anesthesiology.

[39] Khamiees M., Raju P., DeGirolamo A. (2001). Predictors of extubation outcome in patients who have successfully completed a spontaneous breathing trial. Chest.

[40] Aldecoa C., Bettelli G., Bilotta F. (2017). European Society of Anaesthesiology evidence-based and consensus-based guideline on postoperative delirium. Eur J Anaesthesiol.

[41] Canet J., Gallart L., Gomar C. (2010). Prediction of postoperative pulmonary complications in a population-based surgical cohort. Anesthesiology.

[42] Kim J., Kim Y.K., Kim H. (2023). Machine learning algorithms predict successful weaning from mechanical ventilation before intubation: retrospective analysis from the Medical Information Mart for Intensive Care IV database. JMIR Form Res.

[43] Collins G.S., Moons K.G.M., Dhiman P. (2024). TRIPOD+AI statement: updated guidance for reporting clinical prediction models that use regression or machine learning methods. BMJ.

