# Supplementary material for: Phenotype-based modeling of postoperative ventilatory liberation failure: A dynamic mechanistic framework for precision perioperative medicine
Source: J Anesth Transl Med. 2026 Jul 28;5(3):143–51. doi: 10.1016/j.jatmed.2026.06.004 (PMC13449779; doi:10.1016/j.jatmed.2026.06.004)
Supplement: Supplementary file 1 [file mmc1.pdf]

Supplementary

Conceptual Evidence Map

Phenotype-based modeling of postoperative ventilatory liberation failure: A dynamic mechanistic framework for precision perioperative medicine

This conceptual evidence map summarizes how the principal evidence anchors map onto the four mechanistic dimensions of the proposed framework. Consistent with the conceptual-synthesis methodology described in Section 2 of the main manuscript, it is not a systematic or scoping review and does not report pooled effect estimates, a PRISMA flow diagram, or formal risk-of-bias scoring. The working corpus comprised 134 publications identified in PubMed/MEDLINE (from database inception to April 2026, updated May 2026) and supplemented by backward and forward citation tracking, selected for conceptual relevance and synthesized to theoretical saturation. Evidence status is tagged E (evidence-supported) or H (hypothesis-generating, requiring prospective postoperative validation). Reference numbers correspond to the reference list of the main manuscript.

Conceptual domain	Core mechanism	Evidence anchor (ref. nos.)	Evidence origin	Candidate markers	Evidence status	Residual uncertainty
Neuromuscular contractile impairment	Disuse atrophy / VIDD, residual neuromuscular blockade, acquired respiratory-muscle weakness	Diaphragm-protective ventilation and VIDD reviews; ASA/ESAIC neuromuscular-monitoring guidelines; diaphragm-ultrasound meta-analysis; magnetic phrenic-nerve stimulation trial[3,4,9–12,14,15]	ICU mechanistic + perioperative guidelines	Quantitative TOF (recovery), TFdi, MIP, diaphragm excursion/thickness	TOF monitoring/recovery = E; TFdi cut-offs and TOF ≥0.95 target = H	Postoperative value of stricter TOF ≥0.95 and device-based diaphragm preservation unproven
Effort-related diaphragmatic myotrauma	Excessive / dyssynchronous (eccentric) loading; load-induced sarcomere injury	Respiratory-drive and occlusion-pressure physiology; asynchrony and proportional-mode studies; postoperative NAVA RCT[3,15–18,24,25,30]	Mainly ICU physiology; one postoperative RCT	P0.1, Pocc, asynchrony index, accessory-muscle recruitment, dynamic TFdi fall	Asynchrony/accessory-muscle signs = E; numerical cut-offs (P0.1/Pocc, asynchrony index) = H	Harmful postoperative effort thresholds unknown; outcome benefit of proportional modes unproven
Central respiratory-drive suppression	Residual anesthetics/opioids, sedatives, sleep-disordered breathing, metabolic suppression	Capnography and drive physiology; opioid/sedation and OSA literature; nociception-monitor network meta-analysis [16,19,26]	Perioperative + ICU	Rising $EtCO_2$/$PaCO_2$, bradypnea, hypoventilation without distress, sedative burden; nociception-monitor trends (exposure only)	Hypercapnia/bradypnea as hypoventilation signs = E; attribution to central drive and nociception-monitor use as drive markers = H	No validated perioperative phenotype definition separating central suppression from weakness or airway causes
Cardiovascular load intolerance	Weaning-induced pulmonary edema; preload/afterload shift, diastolic vulnerability, congestion	Lung and cardiac ultrasound studies; postsurgical NT-proBNP change; individualized-PEEP trials[20–23,27–29]	ICU weaning + perioperative plausibility	ΔB-lines, BNP/NT-proBNP (ΔNT-proBNP >23.3%), echocardiographic congestion, reactive hypertension	Dynamic B-lines/rising NT-proBNP associated with failure = E (association); ΔNT-proBNP cut-off as stand-alone rule = H	Whether ultrasound/biomarker-guided PEEP prevents WIPO untested; NT-proBNP dynamics need postoperative recalibration

Cross-cutting and non-primary elements. Frailty, sarcopenia, and reduced physiological reserve are treated as baseline modifiers embedded in B_0[13]. Upper-airway obstruction, pharyngeal dysfunction, and airway edema[37,38], secretion burden and ineffective cough[39], and delirium-related behavioral factors[40] are modeled as Stage-3 boundary-condition modifiers rather than primary dimensions. Postoperative pulmonary complications not primarily driven by diaphragm dysfunction are treated as a competing outcome pathway[41]. These elements are excluded from the four primary dimensions because they either lack an independent, separately falsifiable mechanism of respiratory-pump failure or are better represented as determinants of baseline reserve or post-extubation sustainability.

Abbreviations: BNP, B-type natriuretic peptide; $EtCO_2$, end-tidal carbon dioxide; ICU, intensive care unit; MIP, maximal inspiratory pressure; NAVA, neurally adjusted ventilatory assist; NT-proBNP, N-terminal pro-B-type natriuretic peptide; OSA, obstructive sleep apnea; P0.1/Pocc, airway occlusion pressures; PEEP, positive end-expiratory pressure; TFdi, diaphragm thickening fraction; TOF, train-of-four; VIDD, ventilator-induced diaphragmatic dysfunction; WIPO, weaning-induced pulmonary edema.
